# Joint Effects: A Pilot Investigation of the Impact of Bipolar Disorder and Marijuana Use on Cognitive Function and Mood

**DOI:** 10.1371/journal.pone.0157060

**Published:** 2016-06-08

**Authors:** Kelly A. Sagar, M. Kathryn Dahlgren, Megan T. Racine, Meredith W. Dreman, David P. Olson, Staci A. Gruber

**Affiliations:** 1 Cognitive and Clinical Neuroimaging Core, McLean Imaging Center, McLean Hospital, Belmont, Massachusetts, United States of America; 2 Department of Psychiatry, Harvard Medical School, Boston, Massachusetts, United States of America; 3 Department of Psychology, Tufts University, Medford, Massachusetts, United States of America; Chiba University Center for Forensic Mental Health, JAPAN

## Abstract

Marijuana is the most widely used illicit substance in those diagnosed with bipolar I disorder. However, there is conflicting evidence as to whether marijuana may alleviate or exacerbate mood symptomatology. As bipolar disorder and marijuana use are individually associated with cognitive impairment, it also remains unclear whether there is an additive effect on cognition when bipolar patients use marijuana. The current study aimed to determine the impact of marijuana on mood in bipolar patients and to examine whether marijuana confers an additional negative impact on cognitive function. Twelve patients with bipolar disorder who smoke marijuana (MJBP), 18 bipolar patients who do not smoke (BP), 23 marijuana smokers without other Axis 1 pathology (MJ), and 21 healthy controls (HC) completed a neuropsychological battery. Further, using ecological momentary assessment, participants rated their mood three times daily as well as after each instance of marijuana use over a four-week period. Results revealed that although the MJ, BP, and MJBP groups each exhibited some degree of cognitive impairment relative to HCs, no significant differences between the BP and MJBP groups were apparent, providing no evidence of an additive negative impact of BPD and MJ use on cognition. Additionally, ecological momentary assessment analyses indicated alleviation of mood symptoms in the MJBP group after marijuana use; MJBP participants experienced a substantial decrease in a composite measure of mood symptoms. Findings suggest that for some bipolar patients, marijuana may result in partial alleviation of clinical symptoms. Moreover, this improvement is not at the expense of additional cognitive impairment.

## Introduction

Bipolar disorder (BPD), considered one of the most debilitating mood disorders, is the sixth leading cause of disability in the world according the World Health Organization. In those affected, BPD is often a significant source of distress and burden on relatives and caregivers [1]. Further, among Axis I pathologies, BPD carries the highest risk of substance use comorbidity, which can complicate the course of illness and impact treatment outcomes. In fact, patients with co-occurring BPD and substance use often experience poor treatment response, relapse of mood symptoms, psychosocial difficulties, and reduced treatment compliance [2–4]. Despite evidence that suggests substance use is linked to poorer outcomes, some studies have also shown that BPD patients engage in substance use to improve clinical symptoms. Bolton and colleagues [5] found that almost a quarter of those with mood disorders used alcohol or drugs to relieve symptoms, with the highest rates of self-medication seen in bipolar I disorder. In another study, the authors found that specifically amongst BPD patients who use substances, 79% engaged in drug use *specifically* to improve mood symptoms [6].

Marijuana (MJ) is the most commonly used illicit substance in the US; this statistic also holds true among those diagnosed with BPD [7]. Moreover, rates of MJ use disorders in BPD patients have been found to equal or exceed those of alcohol abuse or dependence, particularly in younger patients [8]. Research has also shown that 20–50% of patients report some form of MJ-related problems [9]. In those who endorse MJ-related problems, 63.7% reported disability, as compared to only 44.5% of those not meeting criteria for MJ use disorders, supporting previous findings that patients with BPD who engage in MJ use exhibit reduced compliance, higher levels of illness severity, and increased likelihood to attempt suicide [4, 7–8, 10–12].

While these studies appear to suggest that MJ use *results* in negative outcomes, a specific cause-and-effect relationship has yet to be determined. Although many studies have reported that MJ use precedes the onset of BPD [13–16], it remains unclear whether MJ use contributes to the pathogenesis of the disorder, or if it is used to address symptomatology, perhaps as a form of premorbid self-medication [17–19], especially if traditional pharmacotherapeutic regimens are ineffective at symptom alleviation. Others have also reported that individuals with higher levels of illness severity may be at risk for MJ use *after* the onset of the disorder [20]. Further research is needed to clarify the relationship between MJ use and the manifestation of BPD symptoms. Despite claims of negative outcomes associated with MJ use, whether patients’ view MJ use as successful in symptom improvement is rarely assessed. In a single study of BPD patients, Weiss et al. [21] reported that nearly all patients initiated substance use as the *result* of one bipolar symptom, and the majority of patients reported improvement that was attributable to substance use for at least one symptom. Further, in a review of anecdotal reports of MJ use among BPD patients, the authors concluded that MJ was not utilized for the “high” sought out by recreational users, which may suggest that the effects of MJ are unique in sub-euphoric doses [22]. Regardless of the motive for use, the fact remains that MJ use is common in patients with BPD. As noted above, patients with BPD who use MJ have been shown to have higher illness severity and poorer outcome, yet report subjective improvement in symptoms after using MJ, suggestive of a complex relationship between MJ and mood [23]. Taken together, these data provide evidence that some patients with BPD may derive a clinical benefit from using MJ and highlight the importance of understanding the effects of MJ on mood symptomatology in those diagnosed with affective disorders.

Ecological Momentary Assessment (EMA), utilized in the current study, allows for the investigation of real-time assessment of mood and related symptoms as well as repeated collection of real-time data in participants' natural environment [24–25]. While most symptom assessments and diagnostic tools in both research and clinical settings rely on retrospective recall of emotions and symptomatology using interviews and self-report questionnaires, EMA data is collected in naturalistic, real-world contexts and therefore offers improved ecological validity over traditional, retrospective methods. In fact, retrospective reports of mood have been shown to have a bias towards negative mood states such as anxiety, depression, and helplessness [26]. Collecting data in real-time with EMA reduces error bias from retrospective assessment and limits the effects of recall bias and generalization of symptoms over a period of time (Shiffman et al., 2008). Additionally, in a review article assessing the contribution of EMA on psychopathology research, Myin-Germeys and colleagues [27] suggest that symptoms in psychiatric disorders are dynamic and can meaningfully fluctuate through the course of the day. Thus, the increased ecological validity of EMA tools can provide better insight into the phenomenology and etiology of psychopathology than retrospective techniques. Better understanding of the development, maintenance, and progression of symptoms may lead to improved models of these disorders and help inform future treatment strategies [27].

Given a growing body of research indicating cognitive deficits associated with MJ use, it is also important to explore the impact of MJ across various cognitive domains. Interestingly, MJ users (without Axis I pathology) and BPD patients (who do not necessarily smoke MJ) have been shown to exhibit similar cognitive deficits. MJ use has been linked to impairments across a wide range of areas, including attention [28], memory [29–31], IQ [32–34], and executive function [35–37]. Similarly, BPD patients often evidence cognitive deficits across multiple overlapping domains; in two meta-analyses of euthymic BPD patients [38–39], the authors note marked impairment relative to healthy controls on measures of executive function, verbal memory, and attention. Despite the fact that MJ use and a diagnosis of BPD are both individually related to cognitive deficits, two studies examining neurocognitive function in MJ-smoking patients with BPD report surprising outcomes. Both Ringen et al. [40] and Braga, Burdick, DeRosse, and Malhotra [41] reported a *positive* association between neuropsychological functioning and MJ use in BPD patients, perhaps suggestive of a unique relationship between BPD and MJ use. Specifically, Ringen and colleagues [40] examined a variety of cognitive domains, including psychomotor speed, attention, working memory, executive functioning, and verbal learning. Overall, BPD patients who used MJ demonstrated better performance than patients who did *not* use MJ, although statistically significant results were only observed on tests of executive function. Similarly, Braga et al. [41] reported neurocognitive advantages in MJ-smoking BPD patients, relative to a non-smoking BPD group, spanning several domains, including executive function (Trails B) as well as attention and working memory. These results suggest that despite a more severe clinical course, BPD patients who use MJ may demonstrate a cognitive advantage relative to patients without a history of MJ use, underscoring the need for additional investigation.

Through EMA and a comprehensive neuropsychological battery, the current study aimed to clarify the relationship between acute MJ use and mood symptoms as well as cognitive function in patients with BPD. In order to accurately assess the impact of MJ use, BPD diagnosis, and the additive effects of both MJ use and BPD, we utilized a four-group study design enrolling healthy control subjects without MJ use or Axis I disorders (HC), MJ smokers without other Axis I disorders (MJ), individuals diagnosed with BPD without a history of MJ use (BP), and those diagnosed with BPD who currently used MJ (MJBP). We expected, in line with previous research, that the BP and MJBP groups would have more severely affected mood overall relative to the HC group. However, we further hypothesized that the MJBP group would experience significant mood improvement secondary to MJ use. In addition, although previous studies have shown that MJ use is related to cognitive deficits, there is a relative paucity of literature focused on the association between MJ use and cognitive function in patients with BPD. Therefore, the current study also aimed to determine whether MJ use has a differential effect on cognitive performance in pure MJ smokers, BPD patients who smoke MJ (MJBP), and BPD patients who do not use MJ (BP).

## Materials and Methods

Prior to participation, study procedures were thoroughly explained. All participants were also required to read and sign an informed consent form, a document that describes the procedures, risks, benefits, and voluntary nature of the study. This study and all study procedures were approved by the McLean Hospital Institutional Review Board.

### Participants

As part of a larger study conducted between 2008 and 2014, 21 healthy control subjects (HC), 23 MJ smokers without other Axis 1 pathology (MJ), 18 individuals with bipolar I disorder who do not smoke MJ (BP), and 12 individuals diagnosed with bipolar I disorder who smoke MJ (MJBP), were enrolled and completed neuropsychological assessments. A subset of these participants also completed daily EMA assessments over the course of four weeks to assess mood.

Participants were not enrolled in the current study if they met criteria for any *Diagnostic and Statistical Manual of Mental Disorders* (DSM-IV-TR) Axis I pathology (with the exception of bipolar I disorder in the BPD groups, and MJ abuse/dependence in the MJ-smoking groups), as assessed by the Structured Clinical Interview for DSM-IV, Patient Edition (SCID-P) [42]. Individuals were also excluded if they reported a neurological disorder or significant medical problems, significant head injury with loss of consciousness, or were non-native English speakers (as necessitated by the cognitive assessment battery). Further, no participant was enrolled if they reported more than 15 lifetime uses of any illicit drugs (except MJ for the smoking groups) or recreational use of prescription or over-the-counter (OTC) medications, or had received electroconvulsive therapy.

Subjects enrolled in the MJ and MJBP groups were all well-characterized as chronic MJ smokers who reported smoking a minimum of 2,500 times in their lives, used MJ at least four out of the last seven days and tested positive for urinary cannabinoids. In order to ensure that cognitive test results were not affected by acute intoxication, all participants were also required to abstain from MJ use for at least twelve hours prior to study visits. Upon arrival, all individuals were required to provide a urine sample and, to ensure adherence to the twelve-hour abstinence requirement, were led to believe that this sample could be used to detect use within this time frame. This method has previously been used by our laboratory with success [36, 43–44]. Subjects were assessed for most recent use and any who violated the abstinence schedule or who appeared even vaguely intoxicated were rescheduled for a later date. An aliquot of the urine sample was sent to an outside laboratory for quantification of urinary cannabinoid concentration via gas chromatography–mass spectrometry during the initial and final study visit. Urinary THC levels were averaged across the two study visits.

### Study Design and Measures

After completing diagnostic assessments, subjects who met inclusion criteria were enrolled in the four-week study, which contained a baseline visit and four weekly check-in visits. This study employed a combination Time-Based EMA design and Event-Based Monitoring [24–25]. The Time-Based EMA employed an alarm schedule, which alerted participants to complete three rating sessions per day. Each subject pre-selected three times throughout the day (at least five hours apart), which were tailored to his/her typical daily schedules, to rate their mood. In Event-Based Monitoring, EMA measures are triggered by the occurrence of a specific event. For this study, participants were instructed to complete rating scales as soon as possible after MJ use in order to assess the acute impact of MJ on mood.

At the end of their initial screening study visit, all enrolled participants were issued a Palm Pilot (Palm Tungsten T5 PalmOne PDA) and instructed to use the device to rate their mood three times daily over the course of the four-week study. All individuals rated their mood using a custom-designed application, which contained electronic versions of several clinical rating scales: the Profile of Mood States (POMS) [45], which yields subscores for vigor, anger, confusion, tension, fatigue, depression, and a composite score for total mood disturbance (TMD); the Hamilton Anxiety Rating Scale (HAM-A) [46]; Montgomery-Asberg Depression Rating Scale (MADRS) [47]; and the Young Mania Rating Scale (YMRS) [48]. Participants who smoked MJ were also asked to use the device to record episodes of MJ use to allow for the calculation of pre- and post-MJ use mood changes. More specifically, for each episode of MJ use, participants recorded the amount (in grams), frequency, and mode of MJ use (bong, bowl, joint, etc.). Date and time were automatically recorded at the completion of each scale in order to assist with accurate pre- and post-smoking determinations. However, participants were also given the option to adjust the time of last MJ use when completing post-use ratings. Only ratings identified as being completed within four hours of MJ use were categorized as post-MJ use and used for analysis. To ensure that participants were not arbitrarily answering clinical rating questions, “quality control” questions were interspersed throughout the scales, with such questions such as “who is the current US president?” and “how thoughtfully are you answering these questions?”

In order to establish an estimate of overall intellectual functioning, participants completed the Wechsler Abbreviated Scale of Intelligence (WASI) [49]. In addition, all individuals enrolled also completed a neuropsychological battery designed to assay a variety of cognitive domains. Neuropsychological assessments were typically completed by the end of the first check-in visit, and consisted of a number of measures including the Wisconsin Card Sorting Test (WCST), Trail Making Test, Stroop Color Word Test, the Controlled Oral Word Association Test (COWAT), and Digit Span, which served as direct measures of executive function. The WCST assesses the ability to form abstract concepts, shift and maintain set, and utilize feedback, and is considered a gold-standard measure of executive function [50–51]. The Trail Making Test is comprised of two parts; while Trails A measures visual scanning and psychomotor speed, Trails B serves as a measure of cognitive set-shifting and attention [51]. The Stroop measures the ability to establish competing response tendencies, inhibit inappropriate responses, and resist interference [52]. The COWAT consists of two parts and serves as a measure of phonemic verbal fluency and executive function (participants must generate words starting with the specific letters F, A, and S) as well as verbal memory function (participants are required to generate words from a specific semantic category, in this case “Animals”) [53–54]. The Digit Span subtest from the Wechsler Adult Intelligence Scale—Revised (WAIS-R), requires subjects to recall increasingly longer strings of numbers in forward and then backward order, and reflects attention, working memory and executive functioning [55–56].

Study participants also completed additional cognitive measures, including the California Verbal Learning Test (CVLT), Rey-Osterrieth Complex Figure Test (ROCF), and Hooper Visual Organization Test (HVOT). The CVLT-II requires subjects to learn an orally presented list of words across five trials to assess verbal learning [57]. Errors and clustering strategies (i.e., grouping list items by category) are also documented to assess efficiency of learning. Further, the CVLT incorporates a delay trial, in which individuals are required to remember the list of words after a 20-minute delay in order to assess verbal memory. The ROCF assesses visual-spatial organization as well as visual memory and requires individuals to copy a complex figure and then draw it from memory both immediately and after a twenty-minute delay [51]. Finally, the HVOT, a measure of visuoperception, requires participants to name objects in drawings that have been “cut” into pieces [58].

In addition, study participants completed the Fagerström Test for Nicotine Dependence (FTND) [59] in order to assess current level of nicotine use and level of dependence. The Addiction Severity Index (ASI) [60] was administered to calculate days of alcohol use within the past month. In order to assess average frequency and magnitude of MJ use, a modified timeline follow-back procedure [61] was utilized at weekly study visits, with a specific focus on the past week of use. Participants were asked to report the number of times they smoked MJ, the amount of MJ used (in grams) and the mode of use each time (i.e., joint, blunt, bong, etc.). Lifetime use was also assessed using the SCID-P and guided substance use interviews.

### Statistical Analyses

In order to ensure that groups were well-matched, one-way analyses of variance (ANOVAs) with Scheffé all pairwise post hoc comparisons (two-tailed) were used to compare the four groups on all continuous demographic variables: age, IQ, ASI alcohol use (days/month), and FTND. As analyses identified age as a potential confounding variable, analyses of covariance (ANCOVAs) controlling for age were performed for all comparisons in which age was significantly different between the groups. In addition, chi-squared analyses were used to compare the sex frequencies between the four groups and to compare medication in the BP and MJBP groups. One-way ANOVAs (two-tailed) were conducted to compare age of BP onset in the BP and MJ BP groups, as well as MJ use variables in the MJ and MJBP groups, including age of MJ onset (defined as first *regular* use: a measurable, consistent pattern of use that occurred at least monthly); frequency of MJ use (average number of smoking episodes per week); magnitude of MJ use (average amount, in grams, used each week); duration of use (number of years since onset of regular MJ use); and urinary THC concentration (ng/mL).

### EMA analyses

Average mood scores over the entire four-week EMA data collection period were calculated for all clinical rating scales for each individual. Additionally, for MJ-smoking participants (MJ & MJBP groups), clinical rating scales were coded to indicate whether each rating was collected before (pre) or after (post) MJ use and, with this information, “average pre-MJ use” and “average post-MJ use” ratings scales were calculated for each individual. As previously mentioned, a four-hour threshold was utilized, such that all scales completed within four hours of MJ use were coded as post-MJ use ratings. Ratings completed before MJ use each day, as well as those completed in excess of four hours after MJ use were labeled as pre-MJ use ratings. In an effort to obtain at least one daily baseline rating per day, participants were asked to complete their first set of clinical rating scales prior to smoking MJ. Obtaining overall mood rating averages for each individual, as well as pre- and post-MJ use ratings in the MJ and MJBP participants, provided the opportunity to conduct several levels of analyses in order to assess the unique effects of MJ and BPD on mood, as described below.

One-way ANCOVAs controlling for age were used to analyze differences between the four groups. In order to reduce the number of unnecessary pairwise comparisons, one-tailed Dunnett *t* post hoc comparisons were employed to compare each group to the HC control group. More specifically, to assess the effects of MJ use on both mood and cognition, the HC and MJ groups were directly compared. Similarly, the effect of BPD was examined by comparing the HC group to the “pure” BP group. The additive effect of MJ use and BPD was assessed by comparing the HC group to the MJBP group, and additional one-way ANCOVAs (one-tailed) were also conducted in order to compare the BP group to the MJBP group. These analyses were performed on the overall average mood ratings from the EMA data and, to determine whether significant between-groups differences in overall mood were affected by MJ use, ANCOVAs were repeated using the pre-MJ use average mood ratings, and again using the post-MJ use average mood ratings from the MJ-using groups (MJ and MJBP). In addition, in order to investigate the acute effects of MJ on mood *within* both the MJ and MJBP groups, pre-MJ use average mood scores were compared to post-MJ use average mood scores using paired *t* tests (one-tailed) in each of these two groups separately.

#### EMA compliance analyses and controlling for missing data

Compliance checks were completed during weekly visits and involved saving the EMA data from the PDA to ensure that subjects were completing the majority of their scales. Subjects were informed of their level of compliance at each check-in visit and were encouraged to complete as many scales as possible during the following week. Overall compliance percentages were calculated for each subject. One-way ANOVAs with two-tailed Scheffé all pairwise post hoc comparisons were used to assess compliance percentage differences between the four groups. Additionally, two-tailed Pearson correlations between compliance percentage and EMA average rating scales were used to ensure that missing data did not significantly impact or skew the study findings.

#### Neuropsychological assessment statistical analyses

To examine the effect of BPD on cognition, regardless of MJ use status, one-way two-group ANCOVAs (2-tailed) controlling for age were performed to compare HC participants to participants with BPD (BP and MJBP groups combined). One-way, three-group ANCOVAs with Scheffé all-pairwise post hoc comparisons (2-tailed) were also conducted to compare the HC, BP, and MJBP participants. These three-group analyses assessed the impact of BPD on cognition *exclusive* of MJ use (HC vs BP), and addressed any potential additive effects of MJ use in BP patients (HC vs MJBP and BP vs MJBP).

## Results

### Demographics

Demographics are reported in Table 1. ANOVAs of demographic variables between the four groups revealed that the groups were well matched for IQ and alcohol use (days/month). Between-group differences were noted for age (*F*(3,70) = 5.819, *p* = .001); Scheffé post hoc comparisons indicated that the BP subjects were significantly older than both HC (*p* = .029), and MJ (*p* = .002) participants. Accordingly, age differences were controlled for by utilizing ANCOVAs in all analyses of mood and cognitive performance. Chi-squared analyses indicated that the groups were not well matched for sex (*X*^*2*^(3, *N* = 74) = 11.628, *p* = .009) with the MJBP group having a significantly lower percentage of females than the HC (*X*^*2*^(1, *N* = 33) = 8.972, *p* = .003) and BP groups (*X*^*2*^(1, *N* = 30) = 6.914, *p* = .009). Average scores on the FTND reflected very low nicotine use across the groups. However, between-group differences were noted (*F*(3,70) = 6.335, *p* = .001); the MJBP group reported significantly more nicotine use relative to the HC (*p* = .002), MJ (*p* = .019) and BP (*p* = .004) groups. All of the other groups reported similar FTND scores to one another, and given such low use indicated by the total scores, even for the MJBP group (*M* = 1.92, *SD* = 3.00), it is unlikely that nicotine use was a confound for subsequent analyses.

**Table 1:** ANOVAs for 4-group comparison of demographic data (2-tailed).

Variable	HC	MJ	BP	MJBP	ANOVA		Scheffé All Pairwise Post Hoc Comparisons					
					*F*	*p (η*^*2*^)	HC v MJ	HC v BP	HC v MJBP	MJ v BP	MJ v MJBP	BP v MJBP
*n* Sex	21 (8M, 13F)	23 (16M, 7F)	18 (8M,10F)	12 (11M,1F)	-	-	-	-	-	-	-	-
Age	23.38 *(4*.*20)*	21.96 *(5*.*07)*	28.56 *(6*.*70)*	23.75 *(4*.*45)*	**5.819**	**.001 (.200)**	NS	**.029**	NS	**.002**	NS	NS
IQ	124.65 *(8*.*15)*	119.61*(14*.*35)*	119.17 *(9*.*92)*	115.00 *(9*.*62)*	1.948	.130 (.079)	NS	NS	NS	NS	NS	NS
Alcohol Use (days/month)	5.24 *(5*.*44)*	7.18 *(6*.*05)*	3.94 *(5*.*71)*	5.17 *(4*.*71)*	1.133	.342 (.048)	NS	NS	NS	NS	NS	NS
BPD Age of Onset	-	-	18.06 *(4*.*53)*	15.21 *(3*.*17)*	3.556	.070 (.113)	-	-	-	-	-	-
MJ Age of Onset	-	16.35 *(2*.*31)*	-	16.92 *(2*.*61)*	0.438	.513 (.013)	-	-	-	-	-	-
MJ Smokes/Week	-	15.99 *(7*.*39)*	-	15.55 *(13*.*63)*	0.015	.903 (<.001)	-	-	-	-	-	-
MJ Grams/Week	-	7.21 *(5*.*55)*	-	5.19 *(2*.*76)*	1.289	.265 (.039)	-	-	-	-	-	-
MJ Duration of Use (yrs)	-	5.61 *(3*.*99)*	-	6.83 *(2*.*76)*	0.901	.349 (.027)	-	-	-	-	-	-
Urinary THC (ng/mL)	-	617.53 *(873*.*71)*	-	443.13 *(528*.*43)*	0.370	.547 (.011)	-	-	-	-	-	-

Analyses of medication use revealed that the BP and MJBP groups reported similar medication regimens (Table 2). No significant differences were noted for the frequency of use of different classes of medications: mood stabilizers, antidepressants, antipsychotics, and benzodiazepines. There were also no significant differences between the BP and MJBP groups for the number of medicated vs unmedicated patients within each group. In addition, the BP and MJBP groups were well-matched for age of BPD onset. With regard to MJ use characteristics, the MJ and MJBP groups were also well-matched for age of MJ onset (Table 1), as well as current levels of MJ use. In fact, no significant differences emerged between groups for frequency (smokes/week) and magnitude of MJ use (grams/week), duration of MJ use (years since onset of regular use), or urinary THC levels (ng/mL).

**Table 2:** Chi Squared Analyses of Medication Use in the BP and MJBP Groups.

Variable	BP	MJBP	Chi Squared	
			*X*^*2*^	*p*
Mood Stabilizers	72.22%	75.00%	0.028	NS
Antidepressants	27.78%	16.67%	0.497	NS
Antipsychotics	55.56%	58.33%	0.023	NS
Benzodiazepines	16.67%	8.33%	0.433	NS
Unmedicated	16.67%	8.33%	0.433	NS

*(df = 1,* *n = 30)*

### EMA Results

#### EMA compliance results and controlling for missing data

Across all groups, high levels of compliance were noted for rating scale completion, with the average overall number of completed rating scales at 88% of all possible rating opportunities. Within the HC group, EMA rating compliance indicated that they completed 94% of scales, while the BP completed 90%, and the MJ and MJBP groups each completed 84% of scales. Notably, the MJ and MJBP groups both were required to complete more rating scales than the non-smoking groups, as they rated their mood three times daily *in addition* to completing ratings after MJ use. Therefore, it is not surprising that ANOVA results indicated that despite very high levels of compliance across all groups, some differences in compliance levels were evident (*F*(3,57) = 3.238, *p* = .029). Two-tailed Scheffé all pairwise post hoc comparisons revealed that these differences were driven by a trend in which the MJ group exhibited lower percentages for completion of EMA ratings (*M* = 83.71, *SD* = 9.81) than the HC group (*M* = 93.78, *SD* = 5.91, *p* = .055). Compliance percentages for the BP (*M* = 90.08, *SD* = 9.90) and MJBP groups (*M* = 84.20, *SD* = 19.29) were not significantly different from the other groups. However, due to the significant between-group differences, EMA analyses were re-run with compliance percentage as a covariate, and results remained unchanged. Further, in order to determine the nature of missing EMA data, correlation analyses were also conducted to assess the association between compliance percentage and EMA clinical ratings. Compliance percentage did not significantly correlate with any clinical rating scale (*r*(59)≤.203, *p*≥.12), suggesting that ratings were missing at random, and therefore missing EMA data did not unduly influence clinical mood ratings results.

#### Between-group analyses

Overall average mood ratings from across the 4-week data collection period are presented in Table 3. As expected, compared to the HC group, BP and MJBP participants reported higher levels of anger, confusion, tension, fatigue, depression, and total mood disturbance (TMD) as measured by the POMS, as well as increased anxiety (HAM-A), depression (MADRS), and mania (YMRS). Among patients, those in the BP group reported similar overall mood to MJBP participants, with no significant differences observed on any rating scale, with the exception of higher MADRS scores in the MJBP group. Despite this difference, depression ratings on the POMS were similar between groups, and actually were slightly (albeit not significantly) lower in the MJBP group relative to the BP group. With regard to the MJ group, no significant differences were noted between MJ smokers and HCs for average mood ratings.

**Table 3:** ANCOVAs (controlling for age differences) of the 4-group (HC, MJ, BP, MJBP with Dunnett *t* post hoc comparisons) and 2-group (BP v MJBP) comparisons of overall 4-week average mood EMA ratings (1-tailed).

Variable	HC	MJ	BP	MJBP	4-group ANCOVA		4-group Dunnett *t* Post Hoc Comparisons			2-group BP v MJBP ANCOVA	
					*F*	*p (η*^*2*^)	HC v MJ	HC v BP	HC v MJBP	*F*	*p (η*^*2*^)
*n*	18	21	12	10	-	-	-	-	-	-	-
**POMS**											
Vigor	11.13 *(3*.*77)*	12.92 *(4*.*73)*	9.71 *(3*.*67)*	9.39 *(3*.*23)*	2.039	.060 (.098)	NS	NS	NS	0.357	.279 (.018)
Anger	0.86 *(0*.*76)*	1.31 *(1*.*71)*	4.24 *(3*.*41)*	4.14 *(2*.*59)*	**7.616**	**<.001 (.290)**	NS	**<.001**	**<.001**	0.355	.279 (.018)
Confusion	3.48 *(1*.*44)*	3.51 *(1*.*81)*	7.06 *(3*.*39)*	5.76 *(2*.*69)*	**5.951**	**<.001 (.242)**	NS	**<.001**	**.019**	0.022	.442 (.001)
Tension	3.27 *(1*.*30)*	2.71 *(1*.*61)*	6.92 *(3*.*37)*	6.48 *(3*.*94)*	**7.851**	**<.001 (.296)**	NS	**<.001**	**.003**	0.187	.335 (.010)
Fatigue	3.07 *(1*.*86)*	2.00 *(1*.*65)*	7.06 *(3*.*48)*	5.08 *(2*.*99)*	**8.843**	**<.001 (.321)**	NS	**<.001**	**.050**	0.219	.323 (.011)
Depression	1.31 *(1*.*37)*	1.06 *(2*.*07)*	6.95 *(7*.*04)*	6.61 *(5*.*17)*	**6.462**	**.001 (.257)**	NS	**<.001**	**.002**	0.570	.230 (.029)
TMD	0.86 *(7*.*58)*	-2.33 *(9*.*52)*	22.53 *(21*.*17)*	18.68 *(17*.*41)*	**8.686**	**<.001 (.318)**	NS	**<.001**	**.002**	0.196	.332 (.010)
**HAMA**	0.68 *(0*.*65)*	0.86 *(1*.*23)*	4.43 *(3*.*44)*	4.82 *(3*.*92)*	**9.904**	**<.001 (.347)**	NS	**<.001**	**<.001**	0.771	.120 (.039)
**MADRS**	1.52 *(1*.*37)*	1.57 *(2*.*00)*	7.21 *(5*.*43)*	10.60 *(7*.*01)*	**14.832**	**<.001 (.443)**	NS	**<.001**	**<.001**	**3.183**	**.045 (.143)**
**YMRS**	1.56 *(0*.*84)*	1.50 *(1*.*42)*	3.99 *(3*.*23)*	5.64 *(2*.*83)*	**8.193**	**<.001 (.305)**	NS	**.008**	**<.001**	1.693	.105 (.082)

*POMS = Profile of Mood States, TMD = Total Mood Disturbance, HAM-A = Hamilton Anxiety Rating Scale, MADRS = Montgomery-Asberg Depression Rating Scale, YMRS = Young Mania Rating Scale*.

Analyses of average *pre*-MJ use mood data in the MJ and MJBP group compared to overall average mood in the HC and BP group are presented in Table 4 (top portion of table); analyses of average *post*-MJ mood data in the MJ and MJBP group compared to overall average mood in the HC and BP group are presented in Table 4 (bottom half of table). *Prior to* smoking MJ, the MJBP participants reported higher levels of anger, confusion, tension, depression, and TMD on the POMS, as well as greater anxiety, depression, and mania as measured by the HAM-A, MADRS, and YMRS, relative to the average mood ratings of the HC participants. *After* smoking MJ, while some significant differences remained between the MJBP and HC groups, the MJBP group no longer endorsed significantly higher anger, tension, or TMD on the POMS relative to the HCs. Notably, the MJ participants did not report significantly different mood ratings compared to the HC participants either pre- or post-MJ use.

**Table 4:** Pre- vs Post-MJ Use Mood in MJ and MJBP Participants: ANCOVAs (controlling for age differences) of the 4-group (HC, MJ, BP, MJBP with Dunnett *t* post hoc comparisons) and 2-group (BP v MJBP) comparisons of overall 4-week average mood in the HC and BP participants to average pre- and post-MJ use mood in the MJ and MJBP participants (1-tailed).

Variable	HC (avg)	MJ (pre)	BP (avg)	MJBP (pre)	4-group ANCOVA		4-group Dunnett *t* Post Hoc Comparisons			2-group ANCOVA BP v MJBP	
					*F*	*p (η*^*2*^)	HC v MJ	HC v BP	HC v MJBP	*F*	*p (η*^*2*^)
*n*	18	21	12	10	-	-	-	-	-	-	-
***PRE MJ USE***											
**POMS**											
Vigor	11.13 *(3*.*77)*	13.67 *(5*.*50)*	9.71 *(3*.*67)*	9.26 *(2*.*96)*	**2.792**	**.025 (.130)**	NS	NS	NS	0.672	.211 (.034)
Anger	0.86 *(0*.*76)*	1.29 *(1*.*84)*	4.24 *(3*.*41)*	4.52 *(3*.*14)*	**7.604**	**<.001 (.289)**	NS	**<.001**	**<.001**	0.728	.202 (.037)
Confusion	3.48 *(1*.*44)*	3.00 *(1*.*64)*	7.06 *(3*.*39)*	6.43 *(2*.*96)*	**8.455**	**<.001 (.312)**	NS	**<.001**	**.003**	0.133	.360 (.007)
Tension	3.27 *(1*.*30)*	2.69 *(2*.*12)*	6.92 *(3*.*37)*	7.53 *(4*.*41)*	**8.933**	**<.001 (.324)**	NS	**.001**	**<.001**	1.166	.147 (.058)
Fatigue	3.07 *(1*.*86)*	1.68 *(1*.*70)*	7.06 *(3*.*48)*	4.77 *(3*.*05)*	**9.290**	**<.001 (.332)**	NS	**<.001**	NS	0.464	.252 (.024)
Depression	1.31 *(1*.*37)*	0.71 *(1*.*11)*	6.95 *(7*.*04)*	7.40 *(6*.*06)*	**8.086**	**<.001 (.302)**	NS	**<.001**	**<.001**	0.904	.177 (.045)
TMD	0.86 *(7*.*58)*	-4.30 *(8*.*86)*	22.53 *(21*.*17)*	21.39 *(19*.*68)*	**10.608**	**<.001 (.362)**	NS	**<.001**	**<.001**	0.590	.226 (.030)
**HAMA**	0.68 *(0*.*65)*	0.61 *(1*.*13)*	4.43 *(3*.*44)*	5.06 *(4*.*15)*	**10.997**	**<.001 (.371)**	NS	**<.001**	**<.001**	0.980	.168 (.049)
**MADRS**	1.52 *(1*.*37)*	1.41 *(1*.*63)*	7.21 *(5*.*43)*	11.91 *(7*.*53)*	**18.538**	**<.001 (.498)**	NS	**<.001**	**<.001**	**5.317**	**.017 (.219)**
**YMRS**	1.56 *(0*.*84)*	1.90 *(2*.*44)*	3.99 *(3*.*23)*	6.10 *(3*.*89)*	**7.762**	**<.001 (.294)**	NS	**.020**	**<.001**	2.084	.083 (.009)
***POST MJ USE***											
**POMS**											
Vigor	11.13 *(3*.*77)*	12.02 *(4*.*65)*	9.71 *(3*.*67)*	10.78 *(3*.*52)*	0.656	.292 (.034)	NS	NS	NS	0.095	.381 (.005)
Anger	0.86 *(0*.*76)*	1.43 *(1*.*78)*	4.24 *(3*.*41)*	2.35 *(2*.*54)*	**4.840**	**.003 (.206)**	NS	**<.001**	NS	0.953	.171 (.048)
Confusion	3.48 *(1*.*44)*	3.63 *(1*.*68)*	7.06 *(3*.*39)*	5.85 *(3*.*11)*	**5.771**	**.001 (.236)**	NS	**<.001**	**.017**	0.021	.443 (.001)
Tension	3.27 *(1*.*30)*	2.54 *(1*.*66)*	6.92 *(3*.*37)*	4.15 *(2*.*62)*	**7.707**	**<.001 (.292)**	NS	**<.001**	NS	2.473	.066 (.115)
Fatigue	3.07 *(1*.*86)*	2.77 *(2*.*72)*	7.06 *(3*.*48)*	4.88 *(3*.*24)*	**5.186**	**.002 (.217)**	NS	**<.001**	NS	0.430	.260 (.022)
Depression	1.31 *(1*.*37)*	0.77 *(1*.*23)*	6.95 *(7*.*04)*	4.69 *(3*.*64)*	**6.073**	**.001 (.245)**	NS	**<.001**	**.026**	0.030	.433 (.002)
TMD	0.86 *(7*.*58)*	-0.87 *(10*.*32)*	22.53 *(21*.*17)*	11.15 *(13*.*69)*	**6.626**	**.001 (.262)**	NS	**<.001**	NS	0.511	.242 (.026)
**HAMA**	0.68 *(0*.*65)*	0.82 *(1*.*08)*	4.43 *(3*.*44)*	4.23 *(4*.*12)*	**8.315**	**<.001 (.308)**	NS	**<.001**	**<.001**	0.065	.401 (.003)
**MADRS**	1.52 (1.37)	1.46 (1.71)	7.21 (5.43)	9.35 (6.89)	**12.229**	**<.001 (.397)**	NS	**<.001**	**<.001**	1.341	.131 (.066)
**YMRS**	1.56 *(0*.*84)*	2.30 *(1*.*96)*	3.99 *(3*.*23)*	5.61 *(3*.*81)*	**6.856**	**.001 (.269)**	NS	**.013**	**<.001**	1.091	.155 (.054)

*POMS = Profile of Mood States, TMD = Total Mood Disturbance, HAM-A = Hamilton Anxiety Rating Scale, MADRS = Montgomery-Asberg Depression Rating Scale, YMRS = Young Mania Rating Scale*.

Two-group ANCOVAs directly comparing the BPD patient groups (BP vs MJBP; Table 4) revealed that *prior to MJ* use, MJBP participants exhibited higher depression (MADRS) scores and a trend for higher mania (YMRS) ratings relative to the BP group. Interestingly, *after smoking MJ*, the MJBP group reported decreased levels of depression and mania; MADRS scores fell to a level no longer significantly different from ratings in the BP group, and the trend for higher mania ratings on the YMRS was no longer observed in MJBP participants relative to BP participants. In addition, the MJBP group reported a trend for lower levels of tension on the POMS after MJ use compared to BP patients. Together, these decreases in clinical symptoms led to far lower TMD scores in the MJBP group *after* MJ use, relative to the BP group’s average TMD score (MJBP: 11.15 vs BP: 22.53); however, this difference was not statistically significant.

#### Within-group analyses

**Fig 1:**
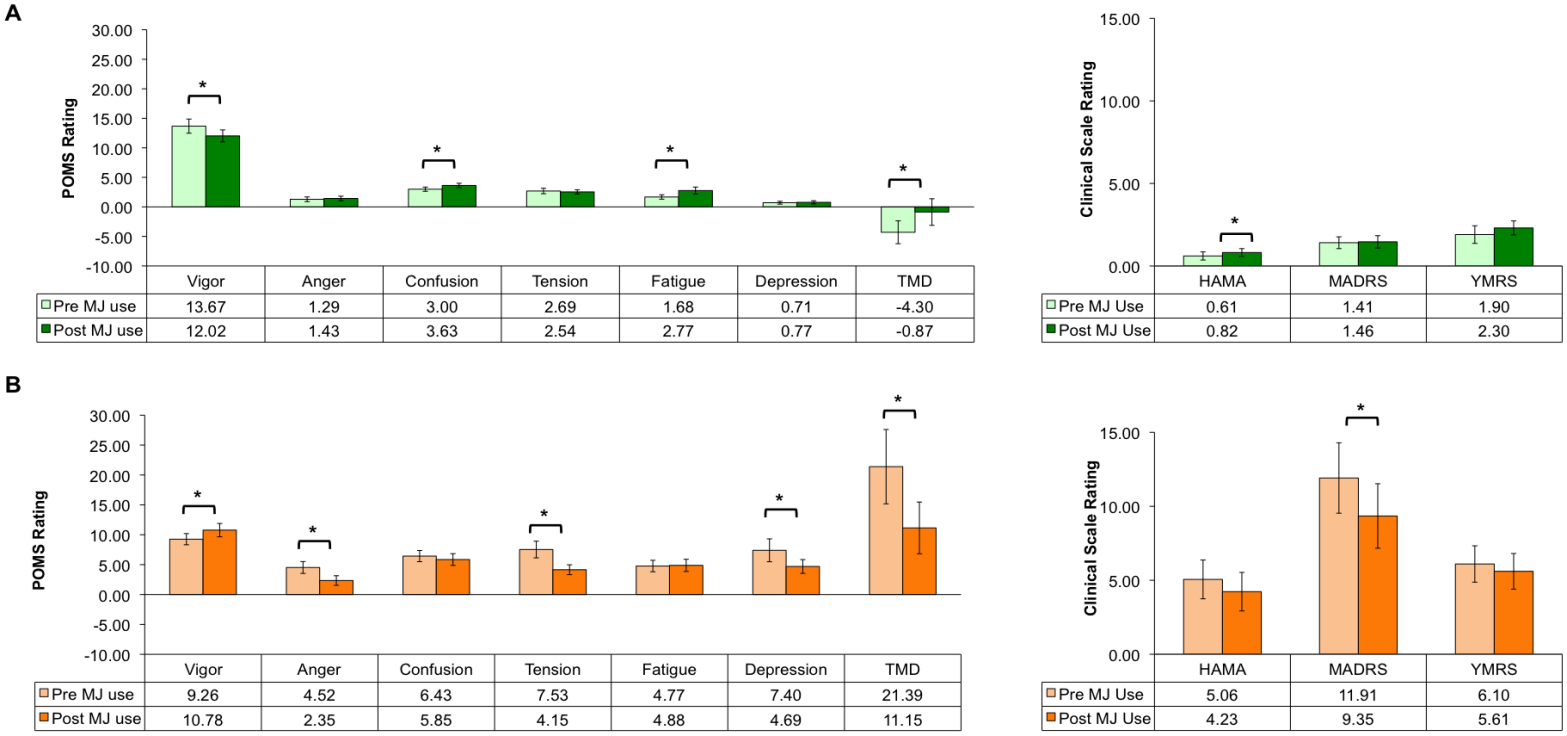
Paired *t*-Test EMA Analyses of Clinical State Pre- versus Post-MJ Use. EMA analyses of clinical state (POMS, HAMA, MADRS, YRMS) changes pre- versus post-MJ use in the (A) MJ group and (B) MJBP group revealed a slight worsening of symptoms in the MJ group after smoking MJ but a significant mood improvement in the MJBP group after smoking MJ, *t(≥9)≥1.942, *p*≤.042, 1-tailed. *POMS = Profile of Mood States*, *TMD = Total Mood Disturbance*, *HAM-A = Hamilton Anxiety Rating Scale*, *MADRS = Montgomery-Asberg Depression Rating Scale*, *YMRS = Young Mania Rating Scale*

Paired *t* tests investigating within-group mood changes pre- and post MJ use suggest that *after* smoking MJ, the MJ smokers experienced slightly worse mood overall. As a group, they reported significantly increased confusion and fatigue, and decreased vigor on the POMS, resulting in higher TMD scores relative to pre-smoking levels. It is of note, however, that their average TMD scores still fell below zero, reflecting very low levels of mood-related symptomatology overall. MJ smokers also reported higher levels of anxiety (HAM-A) *after* MJ use (Fig 1A). In contrast, the MJBP participants reported *positive* changes in mood *after* MJ use. Paired *t* tests comparing pre- and post-MJ use mood ratings within the MJBP group indicated significantly decreased ratings of anger, tension, depression, and TMD scores on the POMS as well as lower levels of depression on the MADRS. In addition, MJBP participants reported increased vigor on the POMS after MJ use (Fig 1B).

### Neuropsychological Assessment

#### HC vs All BP: Effects of BPD on cognition (regardless of MJ use status)

When all BPD patients (BP and MJBP combined) were compared to the HC group, they generally demonstrated poorer performance on tasks of executive function. Specifically, as noted in Table 5, 2-way ANCOVAS revealed that patients with BPD achieved fewer categories, made more perseveration errors and had more losses of set on the WCST. Patients with BPD had significantly longer completion times and made more errors on the Stroop during the Color Naming and Word Reading condition. They also demonstrated slightly slower completion times on the Stroop Interference condition relative to the HCs, although this did not reach statistical significance. Similarly, BPD patients also performed Trails B significantly more slowly and exhibited a trend for more errors on Trails A relative to HCs. BPD patients achieved lower total scores across the three trials of the COWAT in which they had to generate words starting with a given letter (F, A, S); during the semantic category trial (animals) a trend was also observed for fewer words generated among BPD patients. In addition, BPD patients recalled fewer digits on Digit Span overall, including recollection of digits in forward order (Forward) and in reverse order (Backward), which led to lower Total Digit Span scores.

**Table 5:** Neuropsychology Data and Between-Groups Comparisons: ANCOVAs (controlling for age) of the 2-group (HC v All BP) and 3-group (HC, BP, and MJBP) comparisons (2-tailed).

Variable	HC	All BP	BP	MJBP	2-group ANCOVA HC v All BP		3-group ANCOVA HC v BP v MJBP		Scheffé All Pairwise Post Hoc Comparisons		
					*F*	*p (η*^*2*^)	*F*	*p (η*^*2*^)	HC vs BP	HC vs MJBP	BP vs MJBP
*n*	21	30	18	12	-	-	-	-	-	-	-
**WCST**											
Total Categories	9.30 *(0*.*98)*	8.21 *(1*.*26)*	8.29 *(1*.*31)*	8.08 *(1*.*24)*	**9.598**	**.003 (.173)**	**4.858**	**.012 (.178)**	**.041**	**.023**	NS
Total Perseverations	6.40 *(4*.*44)*	10.72 *(7*.*30)*	10.00 *(7*.*16)*	11.75 *(7*.*69)*	**5.718**	**.021 (.111)**	2.996	.060 (.118)	NS	.080	NS
Total Losses of Set	0.15 *(0*.*37)*	0.59 *(0*.*87)*	0.59 *(0*.*87)*	0.58 *(0*.*90)*	**4.694**	**.035 (.093)**	2.318	.110 (.093)	NS	NS	NS
**Stroop**											
Color Naming Time (sec)	49.60 *(7*.*47)*	54.33 *(9*.*01)*	52.17 *(7*.*29)*	57.58 *(10*.*61)*	**4.148**	**.047 (.081)**	**3.444**	**.040 (.130)**	NS	**.038**	NS
Color Naming Errors	0.55 *(1*.*00)*	1.37 *(1*.*33)*	1.67 *(1*.*50)*	0.92 *(0*.*90)*	**5.495**	**.023 (.105)**	**4.811**	**.013 (.173)**	**.021**	NS	NS
Word Reading Time (sec)	38.95 *(4*.*39)*	42.10 *(5*.*82)*	41.56 *(5*.*95)*	42.92 *(5*.*78)*	**6.539**	**.014 (.122)**	**3.201**	**.050 (.122)**	NS	NS	NS
Word Reading Errors	0.35 *(0*.*59)*	0.93 *(1*.*05)*	1.11 *(1*.*08)*	0.67 *(0*.*98)*	**5.041**	**.029 (.097)**	**3.781**	**.030 (.141)**	**.039**	NS	NS
Interference Time (sec)	85.75 *(15*.*60)*	91.63 *(15*.*94)*	89.56 *(16*.*13)*	94.75 *(15*.*82)*	2.763	.103 (.056)	1.435	.248 (.059)	NS	NS	NS
Interference Errors	2.10 *(1*.*94)*	2.03 *(2*.*11)*	2.33 *(2*.*35)*	1.58 *(1*.*68)*	0.046	.831 (.001)	1.172	.319 (.048)	NS	NS	NS
**Trail Making Test**											
A Time (sec)	20.85 *(6*.*29)*	24.07 *(8*.*55)*	23.33 *(6*.*45)*	25.17 *(11*.*24)*	1.919	.173 (.039)	1.160	.322 (.048)	NS	NS	NS
A Errors	0.10 *(0*.*31)*	0.30 *(0*.*53)*	0.39 *(0*.*61)*	0.17 *(0*.*39)*	3.351	.074 (.067)	**3.655**	**.034 (.137)**	NS	NS	NS
B Time (sec)	38.85 *(11*.*21)*	54.23 *(19*.*86)*	55.50 *(21*.*75)*	52.33 *(17*.*38)*	**7.670**	**.008 (.140)**	**3.770**	**.030 (.141)**	**.016**	NS	NS
B Errors	0.15 *(0*.*49)*	0.40 *(0*.*62)*	0.56 *(0*.*70)*	0.17 *(0*.*39)*	0.992	.663 (.004)	1.356	.268 (.056)	.093	NS	NS
**COWAT**											
Total (FAS)	47.65 *(8*.*78)*	39.14 *(11*.*58)*	38.25 *(11*.*69)*	40.20 *(11*.*98)*	**7.318**	**.010 (.158)**	**3.835**	**.030 (.168)**	.060	NS	NS
Semantic Category	26.45 *(6*.*42)*	22.59 *(4*.*82)*	22.25 *(5*.*67)*	23.00 *(3*.*80)*	3.146	.084 (.075)	1.552	.225 (.076)	NS	NS	NS
**Digit Span**											
Forward	9.80 *(2*.*09)*	8.50 *(2*.*35)*	8.24 *(2*.*54)*	8.91 *(2*.*07)*	**4.262**	**.045 (.087)**	2.681	.080 (.109)	NS	NS	NS
Backward	8.85 *(2*.*50)*	7.18 *(1*.*87)*	7.18 *(2*.*24)*	7.18 *(1*.*17)*	**9.792**	**.003 (.179)**	**5.063**	**.010 (.187)**	.077	NS	NS
Total	18.65 *(4*.*06)*	15.68 *(3*.*58)*	15.41 *(4*.*06)*	16.09 *(2*.*81)*	**8.993**	**.004 (.167)**	**5.009**	**.001 (.185)**	**.046**	NS	NS
**ROCF**											
Copy	33.00 *(2*.*92)*	30.93 *(3*.*71)*	31.04 *(4*.*45)*	30.80 *(2*.*79)*	2.491	.123 (.063)	1.325	.278 (.069)	NS	NS	NS
Immediate	22.69 *(7*.*55)*	16.93 *(7*.*30)*	16.83 *(8*.*26)*	17.05 *(6*.*41)*	3.019	.091 (.075)	1.778	.184 (.090)	NS	NS	NS
Delay	22.81 *(6*.*82)*	16.68 *(6*.*58)*	16.63 *(6*.*91)*	16.75 *(6*.*54)*	**4.957**	**.031 (.118)**	2.660	.084 (.129)	.062	.091	NS
**CVLT**											
Trial 1 Correct	8.25 *(1*.*89)*	6.46 *(1*.*91)*	6.88 *(1*.*83)*	5.82 *(1*.*94)*	**8.997**	**.004 (.167)**	**6.005**	**.005 (.214)**	NS	**.005**	NS
Total Correct	61.10 *(8*.*14)*	51.18 *(10*.*53)*	53.35 *(11*.*75)*	47.82 *(7*.*61)*	**10.182**	**.003 (.185)**	**7.192**	**.002 (.246)**	.057	**.002**	NS
Total Perseverations	4.75 *(5*.*44)*	5.57 *(4*.*76)*	5.29 *(4*.*78)*	6.00 *(4*.*94)*	0.331	.568 (.007)	0.212	.810 (.010)	NS	NS	NS
Total Intrusions	1.60 *(2*.*14)*	1.11 *(2*.*74)*	0.53 *(1*.*23)*	2.00 *(4*.*05)*	0.669	.418 (.015)	2.130	.131 (.088)	NS	NS	NS
Total Semantic Clusters	27.10 *(11*.*09)*	18.57 *(13*.*01)*	21.47 *(14*.*82)*	14.09 *(8*.*34)*	**4.393**	**.042 (.089)**	**4.262**	**.020 (.162)**	NS	**.023**	NS
Long Delay Correct	13.90 *(1*.*68)*	10.64 *(3*.*68)*	10.65 *(4*.*14)*	10.64 *(3*.*04)*	**10.328**	**.002 (.187)**	**5.239**	**.009 (.192)**	**.009**	**.024**	NS
**HVOT**											
Total	26.56 *(1*.*76)*	26.41 *(1*.*88)*	25.71 *(2*.*03)*	27.25 *(1*.*34)*	0.016	.901 (<.001)	2.063	.142 (.103)	NS	NS	NS

*WCST = Wisconsin Card Sorting Test, ROCF = Rey-Osterrieth Complex Figure, COWAT = Controlled Oral Word Association Test, CVLT = California Verbal Learning Test, HVOT = Hooper Visual Organization Test*.

On the remaining measures, BPD patients also tended to demonstrate reduced performance. Despite similar scores on the copy condition of the ROCF, they exhibited slightly lower scores on the immediate recall condition, and achieved significantly lower scores on delayed recall relative to the HCs. On the CVLT, BPD patients recalled fewer words during the initial learning trial (Trial 1), as well as across all five trials (Trial 1–5 Total Correct), and after the Long Delay. They also used less semantic clustering on the CVLT across the five trials (Trial 1–5 Total Semantic Clusters). No significant differences in performance were apparent on the HVOT.

#### HC vs BP: Effects of BPD on cognition (exclusive of MJ use)

Post hoc analyses from a three-way ANCOVA comparing HC, BP and MJBP patients revealed that, relative to HCs, the non-MJ smoking BP participants (BP group only) demonstrated similar deficits when examined separate from the MJBP group as when grouped with MJBP participants (see Table 5). The BP group exhibited poorer performance across the majority of assessment measures. Non-MJ smoking BP patients achieved significantly fewer categories on the WCST; they also made more perseverative errors, and had more losses of set, although this did not reach the threshold for significance. Participants in the BP group also made more errors on the Stroop Color Naming and Word Reading subtests. They took significantly longer to complete Trails B, and exhibited a trend for more Trails B errors. Further, they recalled slightly fewer digits across on Digit Span Backwards, which contributed to significantly lower scores on the Total Digit Span scores among BPD patients relative to HCs. On the CVLT, the BP group recalled fewer words than HCs on the CVLT across all five trials and after a delay. On the COWAT and ROCF, although results were not significant, the BP group demonstrated several trends for worse performance, as noted in Table 5. No deficits were noted the HVOT between HCs and the BP group.

#### HC vs MJ: Effects of MJ use on cognition (exclusive of BPD diagnosis)

Similar to our previous report of MJ smokers [43], two-way ANCOVAs directly comparing MJ smokers to HCs demonstrated that pure MJ smokers exhibited impairment on a number of tasks relative to HCs, including the WCST, Trail Making Test, COWAT, and CVLT (See Table 6). Specifically, as noted in the BPD patients, MJ smokers demonstrated poorer executive functioning relative to controls. They achieved fewer categories and made more perseverative errors on the WCST, and took longer to complete Trails B of the Trail Making Test. MJ smokers generated fewer words than HCs on the COWAT when asked to provide words in a given semantic category. On the CVLT, although MJ smokers recalled a similar number of correct words, they had more Intrusions (incorrect responses) and utilized less semantic clustering across trials (Total Semantic Clusters). HC and MJ participants did not differ significantly with regard to their performance of the Stroop Color Word Test, Digit Span, ROCF, or HVOT.

**Table 6:** Neuropsychology Data and Between-Groups Comparisons: ANCOVAs (controlling for age differences) of the 2-group (HC v MJ) comparisons (1-tailed).

Variable	HC	MJ	2-group ANCOVA	
			*F*	*p (η*^*2*^)
*n*	20	23		
**WCST**				
Total Categories	9.30 (0.98)	8.73 (1.55)	**6.746**	**.007 (.147)**
Total Perseverations	6.40 (4.44)	10.50 (8.55)	**12.680**	**.001 (.245)**
Total Losses of Set	0.15 (0.37)	0.36 (0.73)	2.502	.061 (.060)
**Stroop Color Word Test**				
Color Naming Time (sec)	49.60 (7.47)	52.26 (8.43)	1.449	.118 (.035)
Color Naming Errors	0.55 (1.00)	1.09 (1.12)	2.281	.070 (.054)
Word Reading Time (sec)	38.95 (4.39)	39.65 (4.51)	0.256	.308 (.006)
Word Reading Errors	0.35 (0.59)	0.43 (0.59)	0.248	.311 (.006)
Interference Time (sec)	85.75 (15.60)	86.96 (14.50)	0.273	.302 (.007)
Interference Errors	2.10 (1.94)	2.65 (2.25)	0.802	.188 (.020)
**Trail Making Test**				
A Time (sec)	20.85 (6.29)	20.95 (4.53)	0.122	.365 (.003)
A Errors	0.10 (0.31)	0.18 (0.39)	0.231	.317 (.006)
B Time (sec)	38.85 (11.21)	50.50 (22.65)	**4.784**	**.018 (.109)**
B Errors	0.15 (0.49)	0.32 (0.48)	0.863	.180 (.022)
**COWAT**				
Total (FAS)	47.65 (8.78)	48.45 (11.43)	0.035	.426 (.001)
Semantic Category	26.45 (6.42)	21.86 (5.59)	**7.134**	**.006 (.155)**
**Digit Span**				
Forward	9.80 (2.09)	9.78 (2.39)	0.010	.462 (<.001)
Backward	8.85 (2.50)	8.70 (2.24)	0.113	.370 (.003)
Total	18.65 (4.06)	18.48 (3.82)	0.021	.443 (.001)
**ROCF**				
Copy	33.00 (2.92)	32.72 (3.33)	0.206	.327 (.005)
Immediate	22.69 (7.55)	22.42 (7.01)	0.078	.391 (.002)
Delayed	22.81 (6.82)	22.07 (6.65)	0.239	.314 (.006)
**CVLT**				
Trial 1 Correct	8.25 (1.89)	7.39 (2.41)	2.381	.065 (.056)
Total Correct	61.10 (8.14)	57.52 (8.95)	2.717	.054 (.064)
Total Perseverations	4.75 (5.44)	5.57 (5.47)	0.155	.348 (.004)
Total Intrusions	1.60 (2.14)	0.57 (0.90)	**3.637**	**.032 (.083)**
Total Semantic Clusters	27.10 (11.09)	21.61 (8.52)	**4.347**	**.022 (.098)**
Long Delay Correct	13.90 (1.68)	13.09 (3.13)	2.596	.058 (.061)
**HVOT**	26.56 (1.76)	26.80 (2.18)	0.133	.359 (.004)

*WCST = Wisconsin Card Sorting Test,* *ROCF = Rey Osterrieth Complex Figure,* *COWAT = Controlled Oral Word Association Test,* *CVLT = California Verbal Learning Test,* *HVOT = Hooper Visual Organization Test*

#### HC vs MJBP and BP vs MJBP: Potential additive effects of BPD and MJ use

Post hoc analyses from three-way ANCOVAs (HC vs BP vs MJBP) designed to detect potential additive effects of BPD and MJ use revealed that MJBP patients demonstrated some areas of poorer cognitive performance relative to HCs (see Table 5, HC vs MJBP). They achieved fewer categories on the WCST and their performance suggests a trend for making more perseverative errors on this task. On the Stroop, significantly slower times were noted relative to HCs on the Color Naming subtest, while on the CVLT, MJBP participants recalled significantly fewer words during Trial 1, throughout all five learning trials, and after a 20-minute delay. MJBP participants also had fewer semantic clusters on the CVLT. On the ROCF, like the pure BP group, MJBP participants exhibited a trend for lower scores on the Delayed Recall; however, they did not exhibit impairment on the remaining conditions of the task, nor did they demonstrate impaired performance on the Trail making Test, Digit Span, or HVOT relative to HCs.

Although BPD patients and MJ smokers each demonstrated impairment on several measures of cognitive performance relative to HCs, *no differences emerged* when directly comparing the BP and MJBP groups (see Table 5, BP vs MJBP). In fact, Scheffé all pairwise post hoc comparisons revealed no significant between-group differences for BP vs MJBP participants for any task: WCST, Stroop, Trail Making Test, COWAT, CVLT, ROCF, Digit Span, or HVOT.

## Discussion

The current investigation, to our knowledge, marks the first study to examine the effects of MJ on both mood and neuropsychological performance in BPD patients. As the nation explores indications for medical MJ (MMJ), it is imperative to determine how MJ use might affect clinical symptoms in those diagnosed with mood disorders, such as BPD. In addition, given the fact that cognitive decrements are well-documented in both MJ smokers [43, 62–64] and those with BPD [39, 65], it is critical to examine whether these impairments may be exacerbated or possibly ameliorated by the combination of a BPD diagnosis and regular MJ use. Through the utilization of proper control groups (achieved by including four discrete groups: healthy controls, MJ smokers with no Axis I pathology, non-MJ smoking BP patients, and MJ-smoking BP patients), the current study was able to begin to clarify both the individual effects and potential for additive effects of MJ use and BPD on mood and cognition.

As hypothesized, our findings suggest that after smoking MJ, BPD patients experienced improvement in several aspects of clinical state secondary to MJ use. In fact, direct analyses of the MJ-smoking BPD patients (MJBP) *before* and *after* MJ use revealed notable symptom alleviation within four hours of smoking. After smoking MJ, the MJBP group reported significantly lower scores of anger, tension, depression (POMS and MADRS), as well as higher levels of vigor, which led to a marked decrease in TMD scores (22.39 to 11.15), a composite measure of overall mood on the POMS. Further, prior to smoking MJ, the MJBP participants reported slightly worse levels of symptomatology relative to the pure BP group, with higher levels of depressive and manic symptoms. In contrast, after MJ use, the MJBP group demonstrated considerably *lower* levels of tension and lower TMD scores relative to the BP group. In addition, although depression (MADRS) and mania scores were still slightly higher in the MJBP group after MJ use relative to the BP group, scores dropped to levels that were no longer significant or approaching significance between the two groups, highlighting positive changes in mood-related symptoms. In addition, average mood ratings across the course of the study showed that overall mood was comparable between MJBP and BP subjects. Although MADRS scores were generally elevated in MJBP patients, POMS scores for depression were similar between groups (and were actually marginally lower within MJBP participants). As the MADRS reflects specific depressive symptoms, as compared to the POMS which measures self-perceived mood, results may indicate that while certain depressive symptoms were more evident in MJBP participants relative to BP participants, a *self-perceived* mood of depression (i.e., feeling sad, lonely, blue) was not more prevalent in MJBP participants.

To some extent, these findings support recent work, which found that MJ use was correlated with increased positive affect in BPD patients [66]. However, the authors also observed a relationship between MJ use and increased manic and depressive symptoms. Although the authors of this study report both positive and negative fluctuations in clinical symptoms, they posit that bidirectional effects of MJ use, outlined by Ashton and colleagues [22], are likely impacted by a range of factors, including dose, mode of use, and personality differences. In addition, as we learn more about the differential effects of individual constituents of MJ, (i.e., THC vs cannabidiol [CBD]), it is possible that strains higher in certain constituents are at least partially responsible for the moderation of specific dimensions of clinical symptoms. Some research suggests that CBD may be beneficial in alleviating, anxiety, psychosis, and other psychological symptoms [67–70] and may have a pharmacological profile similar to that of antipsychotic medications [69], which are often prescribed to patients with bipolar I disorder. Further, CBD has been shown to be an effective anticonvulsant treatment for those with pediatric seizure disorders [70], another class of drugs frequently prescribed for mood stabilization in patients with BPD.

Interestingly, among pure MJ smokers (those not diagnosed with BPD), beneficial effects on mood were *not* observed in the current study. MJ smokers reported decreases in vigor, as well as higher levels of confusion, fatigue and TMD after smoking, consistent with effects commonly reported in the general population after MJ use. It is of note, however, that MJ smokers continued to exhibit very low levels of mood-related symptoms even after MJ use, suggesting that while their mood did appear to worsen slightly after using MJ, these changes remained far below clinical thresholds. Overall, results may indicate that MJ use may have unique effects in BPD patients, effects which are not necessarily observed in those without Axis I pathology.

With regard to cognitive performance, MJ smokers and BPD patients performed more poorly than HCs overall. However, within the BPD patients, impairment was observed *regardless* of MJ use status; deficits were apparent when the non-smoking BP patients were analyzed as a whole group (BP and MJBP) as well as separately (BP vs MJBP). Overall, patients in both BPD groups demonstrated poorer performance on tasks of executive function. They also exhibited less efficient learning and recall strategies during a serial list-learning task, reduced verbal fluency, inferior attention and working memory, and poorer visuospatial organization. Interestingly, when the non-smoking BP group was compared to the MJBP group, no significant differences across any measure were noted. Taken together, study findings suggest that MJ use may result in at least short-term mood term stabilization for a subset of BPD patients, and further, that MJ use does not have an additive, negative impact on cognitive performance in BPD patients.

These findings provide a valuable contribution to the field, which has only begun to clarify the effects of MJ on mood and cognition in psychiatric populations. While many would posit that the individual relationships between cognitive impairment and both MJ use and BPD would collectively result in a *more severe impact* on cognitive function, some studies have actually reported a cognitive advantage in BPD patients who use MJ regularly [40–41]. In addition, a recent study of MJ-smoking patients diagnosed with schizophrenia found no evidence for an additive effect of MJ use and schizophrenia diagnosis on cognitive dysfunction [71]. In combination, these studies provide evidence that cognitive deficits associated with certain Axis I pathologies may not be worsened by MJ use. In fact, improved cognitive performance may be related to the potential anxiolytic effects of MJ. Anxiety, common in BPD patients [72], often interferes with attention and the ability to encode information, suggesting that if MJ acts as an anxiolytic in at least a subset of patients, this may result in better concentration and enhanced cognitive performance.

Despite these positive changes, one previous study also observed that patients tended to experience improved cognitive performance at the expense of a more severe clinical course [41]. While the current study did not examine long-term treatment outcomes, our preliminary findings provide evidence that BPD patients who smoke MJ may derive at least a short-term clinical benefit. MJBP participants reported improvements in mood within four hours of smoking MJ, did not have elevated average mood ratings (with the exception of the MADRS) relative to the BPD group across the four-week study, and did not experience additional cognitive deficits when compared to the non-smoking BP group. Future studies will need to be conducted in order to investigate the effect of MJ use on clinical course over longer durations of time.

### Limitations

Data from the current study provide critical information about MJ use in psychiatric populations, as guidelines regarding indications for medical MJ are considered; however, these findings must be interpreted in light of several limitations. First, although this study served as a pilot investigation, it is important to acknowledge that the overall sample is moderate in size, with a modest number of patients completing EMA ratings, which may limit the generalizability of findings. For example, only participants who were well characterized as chronic, heavy MJ smokers were enrolled in the current study; participants with less frequent use (i.e., casual MJ smokers) may not experience the same effects of MJ on mood and cognition as observed in this sample of participants. Further, rather stringent enrollment criteria were employed, which excluded participants who reported comorbid diagnoses (via phone interview or through the clinical interview). In addition, participants were required to be predominantly euthymic throughout the course of the study. Although these criteria limited the effects of extraneous variables, the impact of MJ on mood and cognition may differ in those who have been diagnosed with comorbid disorders (i.e., ADHD, PTSD, polysubstance use, etc.) or who may be experiencing more acute clinical symptomatology. Finally, likely related to the geographic region from which patients were recruited (the Greater Boston area is home to many universities, hospitals, and research institutions), participants generally demonstrated higher than average IQs. This may limit generalizability to populations with average to below average cognitive abilities. It will be important for future studies to recruit larger numbers of research participants to further investigate the impact of MJ on mood and cognition in BPD, as well as to examine additional factors that were not explored in the current pilot investigation.

Additionally, although the four groups were not statistically matched for sex (more males were enrolled than females in the MJ and MJBP groups), it is likely that the sex distribution of this sample is actually representative of the larger population. In fact, national surveys of substance-using populations have revealed that males engage in the use of illicit substances, including MJ, more frequently than females [73–74].

Regarding the EMA study design, overall compliance was very high among all study groups with an overall completion rate of 88% of all possible scheduled ratings. Several measures were also put in place to encourage completion of ratings after MJ use, including comparing reported frequency of MJ use during interim visits to the frequency of EMA ratings. However, given the nature of EMA data, it is not possible to guarantee that all participants completed mood ratings immediately after MJ use. In an attempt to address this issue, all participants were asked to adjust the time of last use if necessary, and any ratings reported more than four hours after MJ use were not coded as post-use data. While this four-hour window was selected to capture the acute effects of MJ use on mood, the duration of MJ effects are likely related to a range of factors including, but not limited to the specific product used (i.e. high THC/low THC), amount and frequency of use, mode of use, and metabolism. It may therefore be an important consideration for future studies to explore whether the duration of MJ intoxication is related to the duration of reported symptom improvement by BPD participants.

Further, although this investigation examined the *acute* effects of MJ use in BPD patients, additional investigations should explore the potential long-term impact of MJ use on clinical state. It is of note that over the duration of the study, the overall average mood ratings for the MJBP and BP were not significantly different across any measure (except for the MADRS), which provides preliminary evidence that MJ use may not *directly* result in poorer clinical course. Higher levels of clinical severity previously reported in MJ-smoking BPD patients [4, 8, 11] may be a result of several factors, including a failure to inform clinicians of MJ use. As MJ may partially address mood-related symptoms, the pharmacotherapeutic regimen prescribed by physicians may, as a result, be different from what would normally be prescribed. Additionally, any short-term improvement following MJ use may result in non-adherence with patients’ prescribed medications, which could ultimately result in poorer long-term outcomes.

Due to the preliminary nature of the current study, the relationship between specific patterns or levels of MJ use and symptom improvement were not thoroughly investigated. In addition, all MJ using participants in the current study were chronic MJ users and results may therefore not be generalizable to more casual MJ users. Future investigations should consider the impact of frequency and amount of MJ smoked, as well as mode of use, and strain of MJ used on both cognition and symptomotology. In fact, several studies have shown promise for the alleviation of anxiety using MJ products that contain high levels of CBD [67–68]. Given that CBD is a non-psychoactive phytocannabinoid that has shown promise as an anxiolytic and anticonvulsant (often used to stabilize mood in patients with BPD), high CBD-containing products may afford more viable options than other cannabinoid-based treatments. Therefore, future studies should also aim to explore whether high-CBD relative to low-CBD strains have differential effects in BPD patients as well as other clinical populations.

Finally, it should be noted the current study design does not imply cause and effect, but rather shows a *relationship* between MJ use and mood improvement. Clinical trials will be needed in order to further investigate the potential for MJ and cannabinoid-containing products as a potential treatment for patients with BPD.

## Conclusions

New legislation across the nation has increased the overall accessibility of MJ to the general public for both recreational and medical use. To date, 24 states and the District of Columbia have fully legalized medical marijuana and another 18 states have allowed the use of CBD-based products for medical use. Each state individually regulates the use of MMJ, and perhaps not surprisingly, a wide range of acceptable conditions are often listed as eligible for MMJ certification. While some states include a “catchall” category, allowing physicians to certify conditions at their discretion, other states employ a restrictive list of indications suitable for MMJ. Additional studies are needed to help shape public policy regarding conditions that may be amenable to MMJ treatment, especially with regard to psychiatric illnesses. The current study highlights preliminary evidence that patients with BPD who regularly smoked MJ reported at least short-term clinical symptom alleviation following MJ use, indicating potential mood-stabilizing properties of MJ in at least a subset of patients with BPD. Furthermore, despite previous research showing that MJ use and BPD individually can have a negative impact on cognition, MJ use in BPD patients may *not* result in additional impairment. Further research is warranted to explore the impact of MJ on mood in clinical and non-clinical populations.

## Supporting Information

S1 File Manuscript Database. Demographic, cognitive, and EMA raw data, which were analyzed for the current manuscript, are available and arranged by group (HC, MJ, BP, and MJBP).
(XLSX)
